# Differences in Crenate Broomrape Parasitism Dynamics on Three Legume Crops Using a Thermal Time Model

**DOI:** 10.3389/fpls.2016.01910

**Published:** 2016-12-15

**Authors:** Alejandro Pérez-de-Luque, Fernando Flores, Diego Rubiales

**Affiliations:** ^1^Area de Mejora y Biotecnologia, IFAPA, Centro Alameda del ObispoCórdoba, Spain; ^2^Departamento de Ciencias Agroforestales, ETSI La Rábida, Universidad de HuelvaHuelva, Spain; ^3^Institute for Sustainable Agriculture, Consejo Superior de Investigaciones Científicas (CSIC)Córdoba, Spain

**Keywords:** *Orobanche crenata*, parasitic weeds, GDD, faba bean, lentil, grass pea, legumes

## Abstract

Root parasitic weeds are a major limiting production factor in a number of crops, and control is difficult. Genetic resistance and chemical control lead the fight, but without unequivocal success. Models that help to describe and even predict the evolution of parasitism underground are a valuable tool for herbicide applications, and even could help in breeding programs. Legumes are heavily affected by *Orobanche crenata* (crenate broomrape) in the Mediterranean basin. This work presents a descriptive model based on thermal time and correlating growing degree days (GDD) with the different developmental stages of the parasite. The model was developed in three different legume crops (faba bean, grass pea and lentil) attacked by crenate broomrape. The developmental stages of the parasite strongly correlated with the GDD and differences were found depending on the host crop.

## Introduction

Broomrapes (*Orobanche* and *Phelipanche* spp.) are holoparasitic plants which attach to the host roots and grow at the expense of the host plant's resources. They parasitize a large number of crops, legumes being some of the most severely affected (Joel et al., 2007; Parker, 2013). Because the pathogenesis and most of the growing process take place underground long before diagnosis of the infection, it hampers the development of effective control strategies. In addition, the large amount of seeds released by a single individual (more than 100,000) provides the parasite with a great genetic adaptability to environmental changes (Parker and Riches, 1993; Press and Graves, 1995; Joel et al., 2007). *Orobanche crenata* Forsk. (crenate broomrape) is widely spread through the Mediterranean basin, and remains to date as the most important threat against legumes in the area (Parker, 2009, 2013; Rubiales and Heide-Jørgensen, 2011). Other broomrape species such *Orobanche foetida* Poir. or *Phelipanche aegyptiaca* (Pers.) Pomel can also infect legumes, but are of local importance only.

Although *O. crenata* has a broad host range infecting most legume crops, levels of infection vary between the different species and between accessions within species (Rubiales et al., 2003a,b; Pérez-de-Luque et al., 2004; Román et al., 2007). No complete resistance to *O. crenata* is available in any legume so far, but existing levels of incomplete resistance in some accessions (Rubiales et al., 2006, 2014; Pérez-de-Luque et al., 2007; Fernández-Aparicio et al., 2008, 2012) can delay the growth of already established broomrapes, because the presence of defense mechanisms interferes with the normal development of the parasite (Pérez-de-Luque et al., 2004, 2005).

In addition to the host, the environment can greatly influence the development of the parasitic weed on a given host. Although water availability may be an important factor for broomrape development, particularly in Mediterranean areas (Rubiales et al., 2003a,b; Pérez-de-Luque et al., 2004), it seems that temperature is the main factor affecting broomrape development (Mesa-García and García-Torres, 1986; Castejón-Muñoz et al., 1993; Eizenberg et al., 2004; Ephrath and Eizenberg, 2010). Increased temperature is often associated with an increase in broomrape parasitism, whereas low temperature correlates with lower infections. Cool winters reduce broomrape infection in several crops such as sunflower (*Helianthus annuus* L.), chickpea (*Cicer arietinum* L.), lentil (*Lens culinaris* Med.), pea (*Pisum sativum* L.), vetch (*Vicia sativa* L.) or faba bean (*Vicia faba* L.) (Arjona-Berral et al., 1987; Castejón-Muñoz et al., 1993; Rubiales et al., 2003a,b).

In recent years, several experiments accomplished by Eizenberg and coworkers have correlated the growth of broomrape (*Phelipanche aegyptiaca, O. minor* and *O. cumana*) with the thermal time, measured as growing day degrees (GDD) (Eizenberg et al., 2004, 2005; Ephrath and Eizenberg, 2010; Ephrath et al., 2012). Even more, the thermal time can be used to predict broomrape growth on crop roots under the soil, and mathematical models can be developed based on GDD for optimization of herbicide timing application (Eizenberg et al., 2006). This can be a powerful tool for the chemical control of broomrapes, because one of the main problems to date has been establishing the right time for herbicide applications, as to date no consistent relationships between the growth stages of the different hosts and the parasite development have been found (Arjona-Berral et al., 1987).

However, there are no works to date using a thermal time relation as a tool for showing differences in parasite development on different hosts. If temperature affects both, host and parasite development, a thermal time model could be used as a descriptive method for differences in infection levels integrating the two main factors affecting broomrape development: the host and the temperature. For that reason, the relationship between soil temperature and *O. crenata* growth on three different legume crops has been studied and a putative descriptive model based on work on *O. minor* / *Trifolium* by Eizenberg et al. (2005) is proposed. The hypotheses are that a) there are differences in parasite virulence between different host species, b) a sigmoidal relation between thermal time and parasite development exists and c) such relation will show the differences in parasite development between hosts.

## Materials and methods

### Plant material

Lentils, faba beans and grass peas (*Lathyrus sativus* L.) were included in the study. The three tested species are natural hosts for *O. crenata*, and the cultivars selected (“Rubia de la Armuña” for lentil, “Prothabon” for faba bean, breeding line “ICARDA 2” for grass pea) were susceptible to the parasitic weed attack.

### Field assays

Field trials were conducted over two cropping seasons (2006–2007 and 2007–2008) at Alameda del Obispo experimental station, in Córdoba (southern Spain). The fields located there present a deep loam soil (Typic Xerofluvent). The selected crops were sown by the end of November in a field highly infested with *O. crenata*, in 2 m length rows, with 0.7 m between rows and 20 plants per row. Four plots were set for each crop and every year (12 plots per year), surrounded and delimited by faba bean rows. The experiment was performed as a randomized, complete block design with four replications.

### Temperature data recording

Soil temperature was recorded hourly starting January 1st and using data loggers (Gemini Tinytag Plus Data Logger) buried at 5 cm depth and converted to GDD according to McMaster and Wilhelm (1997) using the following equation:

(1) $$GDD=\sum \left[\frac{T_{max}+T_{min}}{2}-T_{base}\right]$$

where *T*_*max*_ and *T*_*min*_ are the maximum and minimum daily temperatures, respectively, and *T*_*base*_ is the base temperature, all of them measured in degree Celsius (°C). *T*_*base*_ was fixed as 0°C for faba bean and grass pea (Stützel, 1995; Rao and Northup, 2008), and 1.5°C for lentil (Ellis and Barrett, 1994).

### Plant sampling and broomrape development

Beginning the first week of February, five plants were extracted from each crop plot every week. The number of attached broomrapes was recorded and their developmental phase classified according to seven stages (Pérez-de-Luque et al., 2004): Stage 1 (T1), tubercles smaller than 2 mm; Stage 2 (T2), tubercles greater than 2 mm, without root development; Stage 3 (T3), tubercles with crown roots, without shoot formation; Stage 4 (T4), shoot formation, remaining underground; Stage 5 (T5), shoot emergence; Stage 6 (T6), flowering; Stage 7 (T7), setting of seeds.

### Statistical analysis

Broomrape tubercle number was log transformed (log[number of broomrapes per plant]) and the transformed data presented because of nonhomogeneity in variance. In order to model each stage (T1 to T7) of broomrape development, log-transformed broomrape number was nonlinearly regressed to GDD using a three-parameter logistic function (Brown and Mayer, 1988):

(2) $$Y=\frac{\text{a}}{1+{\left(\frac{x}{x_{o}}\right)}^{b}}$$

where *Y* represents log-transformed broomrape tubercle number, *a* represents the upper asymptote (maximum broomrape number), *x* represents GDD, *x*_*o*_ represents the GDD when the *Y* is 50% of maximum, and *b* represents the slope at *x*_*o*_. A *t*-test was used for comparison of the regression parameter estimates in each parasitism stage between crops (Zar, 1999). A *t*-test compares a difference with the standard error of that difference. We used the standard error reported by nonlinear regression. The numerator is the difference between fit values. The denominator is an estimate of the standard error of that difference, computed as the square root of the sum of the squares of the two standard error values. This is a reasonable estimate if the number of data points in the two curves is equal, or nearly so.

## Results

**Figure 1:**
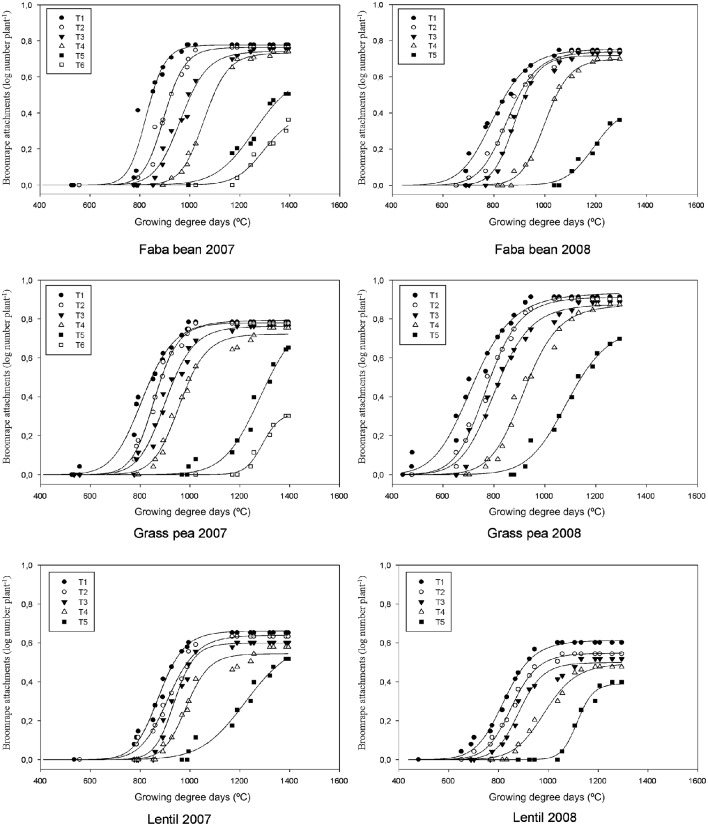
**Three-parameter sigmoid nonlinear regressions between log-transformed broomrape attachment number and growing degree days (GDD) in three crops (faba bean, grass pea and lentil) grouped by broomrape growth stage in two years (2007 and 2008)**.

The combined analysis of variance showed that the interaction between species and year was significant for all the developmental stages of broomrape. A strong relation was found for the broomrape stage and GDD (Figure 1; Tables 1, 2). The model can be divided into three phases for each parasite stage: lag, log and maximum. During the lag phase, the parasite population grows very slowly. After that, the log phase covers an exponential growth of the parasite population. Finally, a maximum is reached and the parasite population remains stable. The lag phase varied slightly with the crop, the parasite stage and the season. For example, the lag phase in faba bean for T3 stage lasted until 850 GDD in 2007 and 750 GDD in 2008; in grass pea until 750 GDD in 2007 and 650 GDD in 2008; and in lentil until 850 GDD in 2007 and 750 GDD in 2008. In general terms, a delay of about 100 GDD was observed in the 2007 season compared to the 2008 season, and the attachments started to develop first in grass pea and later in faba bean and lentil. For that same stage (T3), de log phase ended at about 1150 GDD in 2007 and 1050 GDD in 2008 for faba bean (lasted for about 300 GDD); at 1050 GDD in 2007 and 950 GDD in 2008 for grass pea (lasted for 300 GDD); and at 1050 GDD in 2007 and 950 GDD in 2008 for lentil (lasted for 200 GDD).

**Table 1:** **Coefficients of a three-parameter sigmoid nonlinear regression between log-transformed broomrape number and GDD for season 2006-07**.

**Crop/Parasitism stage**	**Coefficients**						**Regression**	
	***a***	**SE**	***b***	**SE**	***x_0_***	**SE**	**RSME**	***P***
**FABA BEAN**								
T1	0.7769	0.0170	−22.51	3.05	826.41	6.07	0.0561	<0.0001
T2	0.7635	0.0119	−21.75	1.88	897.35	3.88	0.0344	<0.0001
T3	0.7420	0.0167	−18.35	2.06	965.15	6.32	0.0435	<0.0001
T4	0.7330	0.0077	−22.48	1.38	1058.24	4.61	0.0159	<0.0001
T5	0.6425	0.1132	−15.43	3.79	1267.77	36.10	0.0353	<0.0001
T6	0.4020	0.0914	−22.03	6.86	1300.33	35.29	0.0294	<0.0001
**GRASS PEA**								
T1	0.7941	0.0109	−12.89	1.07	811.79	4.73	0.0304	<0.0001
T2	0.7807	0.0074	−18.37	0.96	857.33	2.64	0.0221	<0.0001
T3	0.7631	0.0125	−16.27	1.27	903.04	4.72	0.0339	<0.0001
T4	0.7236	0.0134	−18.02	1.62	958.74	5.21	0.0350	<0.0001
T5	0.8359	0.1606	−16.84	4.07	1288.31	36.30	0.0422	<0.0001
T6	0.3194	0.0233	−37.42	6.65	1291.46	9.31	0.0182	<0.0001
**LENTIL**								
T1	0.6618	0.0098	−17.33	1.31	876.24	4.06	0.0275	<0.0001
T2	0.6406	0.0102	−16.07	1.19	914.13	4.61	0.0271	<0.0001
T3	0.5996	0.0106	−23.50	2.06	937.46	4.39	0.0298	<0.0001
T4	0.5446	0.0125	−21.62	2.98	985.00	6.06	0.0334	<0.0001
T5	0.6304	0.0820	−13.05	2.46	1232.95	31.55	0.0342	<0.0001

*T1, tubercles smaller than 2 mm; T2, tubercles greater than 2 mm, without root development; T3, tubercles with crown roots, without shoot formation; T4, shoot formation, remaining underground; T5, shoot emergence; T6, flowering; a, upper asymptote (maximum broomrape number); x_o_, GDD when the Y is 50% of maximum; b, slope at x_o_; RSME, root mean square error*.

**Table 2:** **Coefficients of a three-parameter sigmoid nonlinear regression between log-transformed broomrape number and GDD for season 2007–08**.

**Crop/Parasitism stage**	**Coefficients**						**Regression**	
	***a***	**SE**	***b***	**SE**	***x_0_***	**SE**	**RSME**	***P***
**FABA BEAN**								
T1	0.7539	0.0126	−12.07	0.89	800.03	5.32	0.0306	<0.0001
T2	0.7387	0.0106	−15.08	1.03	855.39	4.09	0.0254	<0.0001
T3	0.7180	0.0102	−20.14	1.56	883.76	3.71	0.0262	<0.0001
T4	0.6997	0.0108	−21.06	1.26	1001.48	4.18	0.0193	<0.0001
T5	0.4383	0.0855	−21.09	5.64	1196.03	28.9	0.0294	<0.0001
**GRASS PEA**								
T1	0.9343	0.0176	−9.19	0.82	718.41	6.77	0.0419	<0.0001
T2	0.9126	0.0099	−12.24	0.61	774.06	3.54	0.0256	<0.0001
T3	0.8764	0.0187	−11.78	1.08	806.34	6.79	0.0440	<0.0001
T4	0.8725	0.0226	−13.67	1.31	920.03	7.96	0.0400	<0.0001
T5	0.7680	0.0591	−13.71	1.95	1092.56	17.58	0.0376	<0.0001
**LENTIL**								
T1	0.6132	0.0084	−14.11	0.92	823.53	4.05	0.0210	<0.0001
T2	0.5477	0.0061	−16.36	0.92	853.67	3.10	0.0154	<0.0001
T3	0.4993	0.0129	−18.56	2.50	883.62	6.94	0.0319	<0.0001
T4	0.4948	0.0156	−15.68	1.53	987.14	9.05	0.0233	<0.0001
T5	0.3911	0.0163	−35.02	6.22	1116.44	6.24	0.0226	<0.0003

*T1, tubercles smaller than 2 mm; T2, tubercles greater than 2 mm, without root development; T3, tubercles with crown roots, without shoot formation; T4, shoot formation, remaining underground; T5, shoot emergence; a, upper asymptote (maximum broomrape number); x_o_, GDD when the Y is 50% of maximum; b, slope at x_o_; RSME, root mean square error*.

The median (*x*_*o*_) in crenate broomrape parasitism (the GDD in the log phase where the infection was 50% of the maximum) varied also with the crop, the parasitism stage and the season (Tables 1, 2). This index gives an indication about the speed of parasite growth on the host: a low value means that less GDD are needed to reach 50% of the maximum number of attachments, so the parasite grows faster. In general, grass pea presented the lower values, followed by lentil and then faba bean, with some exceptions mainly in season 2007. Broomrape grew faster on grass pea than on the other crops and needed less GDD for developing the shoot (T4, 920–958) than those on lentil (985–987) or faba bean (1001–1058). During season 2007, broomrapes needed some more GDD to reach the median (*x*_*o*_) of all the developmental stages (except T4 in lentil) than during season 2008. Such differences were more accentuated in grass pea (near 100 GDD for most of the stages) than in faba bean or lentil (near 50–60 GDD for most of the stages).

The maximum parasite number was reached in stages T1 to T4 at about 1000 GDD for T1 and T2, 1100–1200 GDD for T3, and 1200 GDD for T4. It was similar for the three crops and the two seasons. However, the number of broomrapes per plant was different depending on the crop: grass pea and faba bean supported a higher number of broomrapes than lentil (Figure 1). In addition, the upper asymptote (or maximum broomrape number, *a*) reached by stages T1 to T4 for each crop was similar between years: 0.7–0.8 (5.0–6.3 broomrapes per plant) for faba bean, 0.7–0.9 (5.0–7.9 broomrapes per plant) for grass pea, 0.5–0.6 (3.2–4.0 broomrapes per plant) for lentil (Tables 1, 2). With respect to the emergence of broomrape (>T5), although the upper asymptote was not reached completely at the end of the experiment for all the crops, the tendency indicates a maximum below the threshold reached by the other stages: 04–0.6 (2.5–4.0 broomrapes per plant) for faba bean, 0.7–0.8 (5.0–6.3 broomrapes per plant) for grass pea, 0.4–0.6 (2.5–4.0 broomrapes per plant) for lentil. Finally, only in faba bean and grass pea during the 2007 season, broomrapes at the T6 stage were detected, with a lag phase at about 1100–1200 GDD, a median (*x*_*o*_) of 1300 and 1292 (respectively), and a maximum of 0.4 (2.5 broomrapes per plant) and 0.3 (2.0 broomrapes per plant) (respectively).

When the model was compared between crops, significant differences were found for the three parameters (*a, b*, and *x*_*o*_) regarding broomrape development (Table 3). The maximum broomrape number (*a*) was significantly different for the underground stages (T1–T4) in lentils respect to the other two crops in 2007 and for the three crops in 2008. For aboveground stage (T5) only grass pea was significantly different from faba bean and lentil in 2008. The median (*x*_*o*_) was significantly different for the three crops considering the underground stages in 2007, except for stage T1 in faba bean and grass pea. In 2008, grass pea values were different from those of faba bean and lentil, except for T1 stage, which values were significant for all the crops. The values for aboveground stages (T5) were significant only in 2008 for faba bean respect to the other two crops. The slope at *x*_*o*_ (*b*) showed the most variable results, with significant differences varying between the season and the parasitism stage considered.

**Table 3:** **Comparison of the coefficients for the three-parameter sigmoid nonlinear regression between the different crops**.

**Parasitism stage**	**Diff. between crops**	**Parameter differences**					
		**Season 2006–2007**			**Season 2007–2008**		
		**a – *P*-value**	**b – *P*-value**	**X_0_ – *P*-value**	**a – *P*-value**	**b – *P*-value**	**X_0_ – *P*-value**
T1	Fb-Lt	0.3989 ^ns^	0.0046^***^	0.0637 ^ns^	0.0000^***^	0.0219^*^	0.0000^***^
	Fb-Ln	0.0000^***^	0.1446 ^ns^	0.0000^***^	0.0000^***^	0.1196 ^ns^	0.0011^***^
	Lt-Ln	0.0000^***^	0.0112^**^	0.0000^***^	0.0000^***^	0.0002^***^	0.0000^***^
T2	Fb-Lt	0.2178 ^ns^	0.1076 ^ns^	0.0000^***^	0.0000^***^	0.0243^*^	0.0000^***^
	Fb-Ln	0.0000^***^	0.0157^*^	0.0080^**^	0.0000^***^	0.3602 ^ns^	0.7395 ^ns^
	Lt-Ln	0.0000^***^	0.1360 ^ns^	0.0000^***^	0.0000^***^	0.0005^***^	0.0000^***^
T3	Fb-Lt	0.3123 ^ns^	0.3962 ^ns^	0.0000^***^	0.0000^***^	0.0001^***^	0.0000^***^
	Fb-Ln	0.0000^***^	0.0857 ^ns^	0.0010^***^	0.0000^***^	0.5959 ^ns^	0.9859 ^ns^
	Lt-Ln	0.0000^***^	0.0055^**^	0.0000^***^	0.0000^***^	0.0203^*^	0.0000^***^
T4	Fb-Lt	0.5749 ^ns^	0.0509^*^	0.0000^***^	0.0000^***^	0.0004^***^	0.0000^***^
	Fb-Ln	0.0000^***^	0.7955 ^ns^	0.0000^***^	0.0000^***^	0.0125^**^	0.1650 ^ns^
	Lt-Ln	0.0000^***^	0.2977 ^ns^	0.0023^***^	0.0000^***^	0.3227 ^ns^	0.0000^***^
T5	Fb-Lt	0.3553 ^ns^	0.8053 ^ns^	0.6952 ^ns^	0.0041^***^	0.2484 ^ns^	0.0044^***^
	Fb-Ln	0.9304 ^ns^	0.5926 ^ns^	0.4743 ^ns^	0.6036 ^ns^	0.1301 ^ns^	0.0285^*^
	Lt-Ln	0.2667 ^ns^	0.4338 ^ns^	0.2621 ^ns^	0.0000^***^	0.0067^**^	0.2217 ^ns^
T6	Fb-Lt	0.3933 ^ns^	0.1268 ^ns^	0.8134 ^ns^	–	–	–
	Fb-Ln	–	–	–	–	–	–
	Lt-Ln	–	–	–	–	–	–

Fb, faba bean; Lt, grass pea; Ln, lentil; T1, tubercles smaller than 2 mm; T2, tubercles greater than 2 mm, without root development; T3, tubercles with crown roots, without shoot formation; T4, shoot formation, remaining underground; T5, shoot emergence; T6, flowering; a, upper asymptote (maximum broomrape number); x_o_, GDD when the Y is 50% of maximum; b, slope at x_o_;

*/**/**: *level of significance at 5, 1, and 0.1% respectively; ns, non-significant*.

## Discussion

The present study confirms that the growth of crenate broomrape on the three tested legume crops is highly temperature-related. The thermal time measured as GDD appears as a valuable tool for describing the parasite growth and establishing the developmental stage of the infection, as previously shown for other crops and broomrape species (Eizenberg et al., 2004, 2005; Ephrath and Eizenberg, 2010; Ephrath et al., 2012).

Although we found differences between years, such differences do not seem to be a problem for developing a descriptive model because, in general terms, they were about 100 GDD or less. Broomrapes growing on grass pea appear as the most affected by year to year variation, with a faster rate and a higher number of infections in the second season (2008) than in the previous one (2007). There are previous reports about differences in broomrape infection depending on the crop and the environmental conditions (Pérez-de-Luque et al., 2004). Such differences could be explained by the uneven rainfall during both seasons. The distribution of the rainfall during both seasons showed some differences (Supplementary Figure 1), with a remarkable higher precipitation during January and April in 2008 compared to 2007, and a low rainfall during March 2008 respect to 2007. The higher rainfall during January 2008 could positively have affected germination of broomrape and the beginning of the infection (allowing a better spreading of germination stimulants), because such conditions have been previously reported as a factor increasing parasitism (Pérez-de-Luque et al., 2004). It is possible that including soil moisture measurements, in addition to thermal time, might improve this kind of models, mainly in dryland fields with no irrigation (highly dependent on rainfall).

Regarding crop species, differences were found in the development of the parasite stages. Most of the parasites evolved from one stage into the next one (T1 to T2, etc.). However, the upper asymptote (*a*) (the maximum) was lower as the developmental stage advances. In the T5 stage emergence differences between crop species become more marked: a lower proportion of broomrapes emerged in faba bean compared with the other two crops. It seems that broomrapes attached to grass pea or lentil can evolve more easily into more advanced developmental stages compared to faba bean, which could be explained by a better availability of resources from such host. It is possible that severity of the attack and the development of broomrapes could be related to physiological traits of the host plant determining the allocation of nutrients into the parasite, as has been shown in *Striga* spp. (Arnaud et al., 1999). In addition, the size of emerged broomrapes on faba bean was bigger than those on grass pea and lentil. As big individuals are more competitive for resources than small ones, intra-specific competition might play a role here.

The comparison of the descriptive thermal model between the three crops confirms significant differences regarding the evolution of the developmental stages of broomrape. The most consistent differences are found when the parameters (mainly *a* and *x*_*o*_) of the underground stages (T1-T4) are considered. The thermal models are usually associated to a specific host (Eizenberg et al., 2004, 2005; Ephrath and Eizenberg, 2010; Ephrath et al., 2012) and they link phenological events with temperature (GDD). This means that the models include physiological traits of the host (for example, taking into account the crop's base temperature). Considering this, differences in the parameters of the model for different crops are pointing out differences in the virulence of the parasite against each crop. In the same way, such differences could be used for differentiating susceptible from resistant genotypes: resistant accessions have been shown to delay broomrape development in the field (Rubiales et al., 2003a; Pérez-de-Luque et al., 2004). Additionally, resistance to broomrape has been reported as temperature dependent in some cases (Eizenberg et al., 2003), so a model considering the environmental temperature would be desirable for evaluating sources of resistance in a breeding programme. Future work should involve validation of this kind of descriptive model in order to develop a predictive one, using different locations and cultivars with different degrees of resistance against crenate broomrape, in order to use it as a tool for plant breeding.

## Author contributions

AP and DR: Conceived and designed the experiments; AP: Developed the experiment; FF performed the statistical analysis; AP, FF, and DR: Discussed and interpreted the results; AP: Wrote a first draft of the manuscript and DR and FF helped with the writing and further edition and correction process; DR: Contributed materials, equipment, and analysis tools.

### Conflict of interest statement

The authors declare that the research was conducted in the absence of any commercial or financial relationships that could be construed as a potential conflict of interest.
