# Supplementary material for: Differences in Crenate Broomrape Parasitism Dynamics on Three Legume Crops Using a Thermal Time Model
Source: Front Plant Sci. 2016 Dec 15;7:1910. doi: 10.3389/fpls.2016.01910 (PMC5156954; doi:10.3389/fpls.2016.01910)
Supplement: Supplementary file 1 [file Image1.PDF]

Supplementary Material

Differences in crenate broomrape parasitism on three legume crops using a thermal time descriptive model

Alejandro Pérez-de-Luque*, Fernando Flores, Diego Rubiales

*** Correspondence:** Corresponding Author: alejandro.perez.luque@juntadeandalucia.es

1 Supplementary Figures

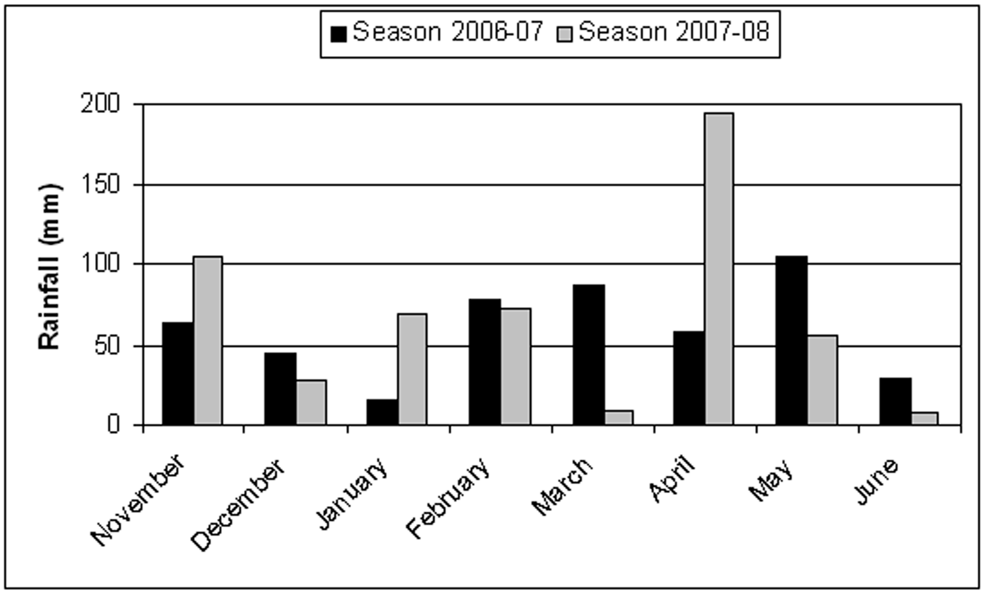

Supplementary Figure 1. Monthly rainfall (mm) during the seasons 2006-2007 and 2007-2008 at the experimental fields in Córdoba, Spain.
